# DNA Methylation Correlates With Responses of Experimental *Hydrocotyle vulgaris* Populations to Different Flood Regimes

**DOI:** 10.3389/fpls.2022.831175

**Published:** 2022-03-07

**Authors:** Mo-Zhu Wang, Hong-Li Li, Min Tang, Fei-Hai Yu

**Affiliations:** ^1^Institute of Wetland Ecology & Clone Ecology/Zhejiang Provincial Key Laboratory of Plant Evolutionary Ecology and Conservation, Taizhou University, Taizhou, China; ^2^State Key Laboratory of Systematic and Evolutionary Botany, Institute of Botany, Chinese Academy of Sciences, Beijing, China; ^3^School of Ecology and Nature Conservation, Beijing Forestry University, Beijing, China

**Keywords:** artificial populations, epigenetic variation, clonal plant, phenotypes, *Hydrocotyle vulgaris*, flooding

## Abstract

Epigenetic mechanisms such as DNA methylation are considered as an important pathway responsible for phenotypic responses and rapid acclimation of plants to different environments. To search for empirical evidence that DNA methylation is implicated in stress-responses of non-model species, we exposed genetically uniform, experimental populations of the wetland clonal plant *Hydrocotyle vulgaris* to two manipulated flood regimes, i.e., semi-submergence vs. submergence, measured phenotypic traits, and quantified different types of DNA methylation using MSAP (methylation-sensitive amplified polymorphism). We found different epi-phenotypes and significant epigenetic differentiation between semi-submerged and submerged populations. Compared to subepiloci (denoting DNA methylation conditions) for the CG-methylated state, unmethylation and CHG-hemimethylation subepiloci types contribute more prominently to the epigenetic structure of experimental populations. Moreover, we detected some epimarker outliers potentially facilitate population divergence between two flood regimes. Some phenotypic variation was associated with flood-induced DNA methylation variation through different types of subepiloci. Our study provides the indication that DNA methylation might be involved in plant responses to environmental variation without altering DNA sequences.

## Introduction

Plants exposed to environmental changes often exhibit plastic phenotypes (Putnam et al., 2016; Colicchio et al., 2018). The classical view advocates that an individual phenotype is determined by its environment, genotype, and their interaction, including both plasticity and evolutionary adaptation (Richards et al., 2010; Bian et al., 2013). However, epigenetic regulation (e.g., DNA methylation, histone modifications, chromatin remodeling, and expression of non-coding RNAs) without changing DNA sequence has been widely considered as another candidate mechanism accounting for plant phenotypic variation (Bossdorf et al., 2008; Marfil et al., 2009). Nowadays, the best-studied hallmark of epigenetic modification is DNA methylation, which is mainly the addition of a methyl group to the C5 position of a cytosine residue in three different sequence contexts (CG, CHG, and CHH sites, *H* = A, C, T), *via* catalysis of several DNA methyltransferase enzymes (Bossdorf et al., 2008; Zoldoš et al., 2018).

Alteration of epigenetic markers mainly originate from genetic variation, environmental induction, or spontaneous epimutations (Dubin et al., 2015; Trucchi et al., 2016; Richards et al., 2017). According to dependence degree on genetic context, epigenetic variation could be classified into three categories: obligate (fully dependent), facilitated (semi-independent), or pure (completely independent) (Richards, 2006; Robertson and Richards, 2015). Unlike genetic variation, DNA methylation patterns are sensitive to changing environments and commonly possess a much higher variation rate (Schulz et al., 2014; Jueterbock et al., 2020). Such variation can be reversibly transient within one generation or stably heritable to several generations (Angers et al., 2010; Paun et al., 2010; Colicchio et al., 2018). DNA methylation alters gene expression through transcriptional repression or remodeling chromatin, further affecting plant phenotypes (Boyko and Kovalchuk, 2010; Grativol et al., 2012; Griffin et al., 2016; Colicchio and Herman, 2020). Therefore, environment-induced epigenetic regulation could not only offer a rapid pathway for phenotypic plasticity, but also underlie plant adaptive evolution when across-generational plasticity confers fitness benefits in predictable environments (Putnam et al., 2016; Huang et al., 2017; Groot et al., 2018; Colicchio and Herman, 2020).

Depending on sequence context, different DNA methylation types (i.e., CG/CHG/CHH) vary in their responses to environmental factors, associations with genetic variation, or functions in gene expression, etc. (Schulz et al., 2014; Colicchio et al., 2018). For instance, in *Arabidopsis thaliana* accessions, CHH methylation of transposable elements (TEs) was sensitive to growth temperature and under *cis*- and *trans*-acting genetic control, whereas CG methylation on the gene coding regions was independent of genetic effects and instead strongly correlated with the latitude of origin (Dubin et al., 2015). In general, CG methylation in gene bodies (GbM) usually activates gene expression, while that in promoters and TEs is associated with gene silencing (Dubin et al., 2015; Colicchio et al., 2018; Jueterbock et al., 2020). Non-CG methylation (i.e., CHH or CHG methylation) mostly occurring in transposons or repeat regions seems to regulate transcriptional repression through chromatin remodeling (Grativol et al., 2012; Schulz et al., 2013; Dubin et al., 2015; Colicchio et al., 2018).

To explore ecological and evolutionary significance of DNA methylation, the first step is to find evidence that at least part of the epigenome changes correlate with plant stress-responses. In recent years, there are accumulating ecological studies exploring different DNA methylation types responding to environmental stresses and/or their relations to plant phenotypic characteristics. Most of these studies focused on plants with the genome reference at the individual level (e.g., Colicchio et al., 2018; Jueterbock et al., 2020). However, roles of epigenetic variation in plastic responses of natural populations to specific environment changes is still largely unknown, especially for non-traditional model organisms lacking the genome reference (Paun et al., 2010; Abratowska et al., 2012; Rico et al., 2014; Watson et al., 2018).

Given the sensitivity of DNA methylation to environmental variation, a direct test for the epigenetic contribution in natural systems could be confounded by complex and dynamic natural conditions (Schulz et al., 2014). Moreover, some previous studies used genetically diverse plant materials, which might hardly disclose the pure epigenetic effects with the presence of genetic variation (Lira-Medeiros et al., 2010; Schulz et al., 2014; Robertson et al., 2017). Therefore, manipulated experimental populations without any genetic variation, such as those consisting of genetically identical asexual individuals (ramets) vegetatively propagated by a single genet (clone) of clonal plants, are better materials to strictly assess roles of epigenetic variation in plastic responses (Rapp and Wendel, 2005; Verhoeven et al., 2010; Verhoeven and Preite, 2014; Huang et al., 2017).

The wetland clonal plant *Hydrocotyle vulgaris* L. (Araliaceae) is considered potentially invasive in China due to high phenotypic plasticity, rapid clonal growth, strong adaptability, and exclusion of other native species (Miao et al., 2011; Liu et al., 2014; Dong et al., 2015). Our previous study showed that the natural *H. vulgaris* populations in southern China possessed low genetic variation but high epigenetic variation, and that their phenotypic variation was largely correlated with epigenetic variation rather than genetic variation (Wang et al., 2020). *H. vulgaris* often experiences water depth changes, which may represent a strong selective force for its population diversification (Wang et al., 2020). In this study, we explored roles of different DNA methylation types in phenotypic responses of *H. vulgaris* to flood variation by exposing its genetically uniform experimental populations to two different flood regimes and by evaluating phenotypic and DNA methylation consequences using MSAP (methylation-sensitive amplified polymorphism). Specifically, we addressed the following questions. (1) What are the phenotypic responses of experimental *H. vulgaris* populations to different flood regimes? (2) What DNA methylation patterns can be generated in different flood regimes? (3) Are environmentally induced alterations in different methylation types related to phenotypic variation?

## Materials and Methods

### Material Propagation

From June to August 2016, 128 plants of *H. vulgaris* were collected from 10 natural populations in southern China (Wang et al., 2020). Using AFLP (amplified fragment length polymorphism), we distinguished 20 genotypes from the 128 individuals, among which a single wide spread genotype accounted for 82% of the total samples and dominated in all 10 populations (Wang et al., 2020). Plants of the most dominant genotype were mixed cultivated and vegetatively propagated under the same condition in a greenhouse at Taizhou University. In early July 2017, we selected more than 576 newly generated similar-sized ramets at the same developmental stage. Each ramet consisted of one node, one leaf and some adventitious roots (petiole length: 22.5 ± 0.2 cm, mean ± SE, *n* = 30). To ensure that all ramets are epigenetically uniform at the start of the experiment, thirty ramets of them were randomly selected for detection of DNA methylation patterns by MSAP, and were identified to be assigned to the same epigenotype.

### Experimental Design

The remaining 546 ramets were used for the experiment described below. We constructed experimental populations of *H. vulgaris* in six big plastic tanks (1.38 m in bottom diameter, 1.60 m in top diameter and 0.89 m in height) filled with a 30-cm-deep mixture of sand and local soil at a 1:1 volume ratio. In each tank, the 91 similar-sized, genetically identical ramets were evenly planted in the range of a 50-cm-edged hexagon from the center point of the soil surface, with two adjacent ramets spacing 10 cm apart (Supplementary Appendix 1A). The soil in the tanks was always kept moist after planting. After 20 days recovery of the six established experimental populations, two flooding treatments were applied. No ramet died before the flooding treatments.

The two flooding treatments were semi-submergence and submergence, each with three replicate tanks (experimental populations). For the semi-submergence treatment, the tank was filled with tap water to a depth of 10 cm above the soil surface, so that the ramets could protrude from the water surface. By contrast, for the submergence treatment, the water level in the tank was maintained 30 cm above the soil surface, so that the ramets were submerged (under the water surface). The experiment was conducted in an open area at Taizhou University, and all the six tanks were placed closely and randomly to avoid potential confounding effects of micro-environmental differences.

### Harvest and Measurements

The experiment lasted from 10 September to 20 December 2017. At harvest, we uniformly set 19 sampling points in each tank, similar to the planting approach, and the difference was that the sampling points were at 25 cm intervals (Supplementary Appendix 1B). At each sampling point, we took two connected mature ramets with fully expanded leaves: one ramet was randomly selected for phenotypic measurement and the other for epigenetic analysis.

For epigenetic analysis, the leaf of the ramet was dried in silica gel. Total genomic DNA from 30 mg of the dry leaf was extracted using Dingguo Plant Genomic DNA Kit (Beijing, China), and quantified spectrophotometrically. After verifying integrity and purity by 1% agarose gel electrophoresis, DNA was diluted to 20 ng/μL as the starting material for epigenetic analysis. The MSAP protocol and scoring method were exactly the same as our previous study, with five selective primer combinations, i.e., E-AGT/H-TAT, E-AGT/H-TTC, E-ATC/H-TGA, E-AAC/H-TCG, and E-ATG/H-TGA (Wang et al., 2020). The error rates for *Hpa*II and *Msp*I scores were about 0.65 and 0.46%, respectively (Wang et al., 2020). Only the repeatable markers were involved in the following molecular analyses.

To quantify phenotypic responses, we first measured leaf petiole length, leaf area and stem internode length of the ramet. Then, the petiole, leaf blade and stem internode were dried at 90°C for 48 h and weighed. Specific petiole length was calculated as petiole length per unit petiole dry mass, specific leaf area as leaf area per unit leaf dry mass, and specific internode length as internode length per unit internode dry mass.

### Data Analysis

We used nested ANOVA to test the effect of flooding treatments on each of the six phenotypic traits (petiole length, specific petiole length, leaf area, specific leaf area, internode length, and specific internode length) at the population level. Experimental populations were nested within the treatment. Before analyses, specific petiole length, internode length and leaf area were log-transformed to improve homoscedasticity (Supplementary Appendix 2).

For MSAP data, the presence or absence of the bands from specific isoschizomer digestions (*Eco*RI/*Hpa*II and *Eco*RI/*Msp*I) results in four conditions of a particular fragment: (I) bands present in both enzyme combinations (1/1), indicating an unmethylated state; (II) bands absent in both enzyme combinations (0/0), indicating an uninformative state; (III) bands present only in *Eco*RI/*Msp*I profiles (0/1), indicating hemi- or fully methylated CG-sites; (IV) bands present only in *Eco*RI/*Hpa*II profiles (1/0), indicating hemimethylated CHG-sites. Due to the fact that different methylation states participate in different regulating processes, considering them separately would give the most comprehensive picture of DNA methylation (Schulz et al., 2013). Therefore, we used the “Mixed-Scoring 2” approach implemented in R script “MSAP_calc.r” (Schulz et al., 2013) to transform the three discernible methylation status represented by combination of *Eco*RI/*Hpa*II and *Eco*RI/*Msp*I banding patterns at each epilocus into binary matrices of different types of subepiloci. Thus, for each epilocus, up to three subepiloci can be generated: u-subepilocus (denoting the unmethylated loci where type I is scored as 1 and other types were scored as 0), m-subepilocus (denoting the CG-methylated loci where type III is scored as 1), and h-subepilocus (denoting the CHG-hemimethylated loci where type IV is scored as 1) (Schulz et al., 2014).

Based on the binary matrices of all MSAP subepiloci and each subepiloci type, we conducted the following analyses. Epigenetic diversity of each experimental population in terms of the percentage of polymorphic loci (*PLP*) and Shannon’s information index (*H*) were assessed by “MSAP_calc.r” (Schulz et al., 2013). To visualize population epigenetic structure, principal coordinate analyses (PCoA) were performed with GenALEx 6.5 based on the matrix of Nei’s distances (Peakall and Smouse, 2012). Epigenetic differentiation at different hierarchical components, that is, between treatments (ϕ_*RT*_), among experimental populations within treatments (ϕ_*PR*_) and within experimental populations (ϕ_*PT*_), was calculated using analysis of molecular variance (AMOVA) with Genalex 6.5. Significance levels were determined after 9,999 permutations.

To identify putatively adaptive epiloci that may facilitate shaping population epigenetic responses to flood variation, we performed outlier detection based on individuals from different experimental populations by using the BayeScan 2.1 (Foll and Gaggiotti, 2008) for estimating the posterior odds (PO) of each epilocus. The analyses were run for 100,000 iterations, with a burn-in of 50,000 iterations, a sample size of 5,000 and a thinning interval of 10. An additional burn-in was carried out by 20 short pilot runs of 5,000 iterations. Only loci exceeding a “strong” detection level [log_10_ (PO) > 1] were considered as putative outliers.

To establish the relationships between environmental, epigenetic and phenotypic variation, structural equation modeling (SEM) was conducted in AMOS 24.0, by relating flood regime and epigenetic variation on phenotypic variation. For each subepiloci type, we examined the direct effects of methylation variation (first three PCoA axis for corresponding subepiloci) and the environmental factor (two flood regimes; semi-submergence was coded as “0,” while submergence treatment was coded as “1”) on phenotypic variation (six phenotypic traits), and indirect effects of flood regimes on phenotypic traits through methylation variation.

## Results

### Phenotypic Responses to Different Flood Regimes

**FIGURE 1:**
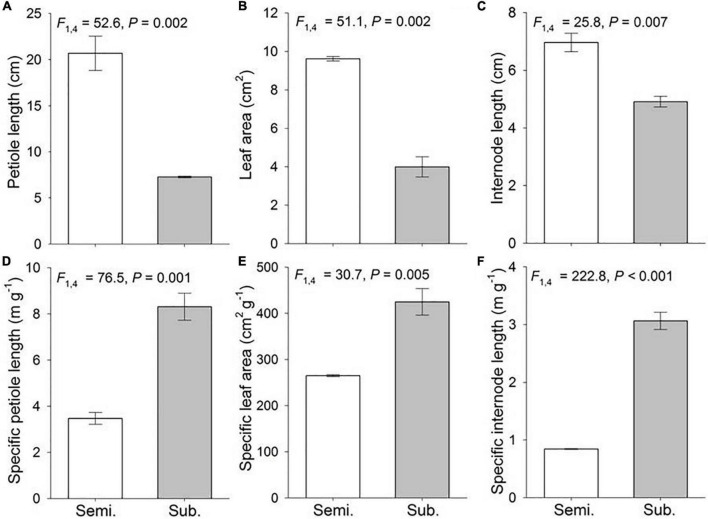
Phenotypic traits of the *Hydrocotyle vulgaris* at population level under semi-submergence (Semi.) and submergence (Sub.). Phenotypic traits are **(A)** petiole length, **(B)** leaf area, **(C)** internode length, **(D)** specific petiole length, **(E)** specific leaf are, and **(F)** specific internode length. Mean ± SE are shown (*n* = 3). *F*, *P*, and degree of freedom of nested ANOVAs are also given.

Flooding significantly affected phenotypic traits of *H. vulgaris* (Figure 1). Submerged populations exhibited significantly shorter petiole length, internode length and smaller leaf area, but higher specific petiole length, specific internode length and specific leaf area than semi-submerged populations (Figure 1).

### Epigenetic Responses to Different Flood Regimes

#### Epigenetic Diversity

The MSAP analysis resulted in 345 scorable epiloci, of which 75 (21.7%) were polymorphic. Mixed scoring 2 detected 144 polymorphic subepiloci, including 66 u-, 42 m-, and 36 h-subepiloci. Epigenetic diversity of submerged populations was significantly higher than that of semi-submerged populations, as quantified by percentage of polymorphic loci and Shannon’s information index based on all subepiloci, u-subepiloci and m-subepiloci (Table 1 and Supplementary Appendix 3).

**TABLE 1:** Epigenetic diversity of the six clonally propagated populations of *Hydrocotyle vulgaris* under semi-submergence (Semi.) and submergence (Sub.) as quantified by **(A)** percentage of polymorphic loci and **(B)** Shannon’s information index based on all subepiloci and u-, m-, and h-subepiloci of MSAP.

	All subepiloci		u-subepiloci		m-subepiloci		h-subepiloci	
	Semi.	Sub.	Semi.	Sub.	Semi.	Sub.	Semi.	Sub.
**(A) Percentage of polymorphic loci (*PLP*)**								
Replicate 1	9.03	45.14	4.55	62.12	11.90	42.86	13.89	16.67
Replicate2	10.42	51.39	6.06	39.39	14.29	61.90	13.89	61.11
Replicate3	16.67	38.19	12.12	46.97	21.43	35.71	19.44	25.00
Mean	**12.04**	**44.91**	**7.58**	**49.49**	**15.87**	**46.82**	15.74	34.26
**(B) Shannon’s information index (*H*)**								
Replicate 1	0.049	0.282	0.024	0.391	0.077	0.269	0.061	0.098
Replicate 2	0.069	0.296	0.039	0.255	0.106	0.372	0.079	0.281
Replicate 3	0.101	0.237	0.087	0.326	0.122	0.200	0.103	0.116
Mean	**0.073**	**0.271**	**0.050**	**0.324**	**0.102**	**0.280**	0.081	0.165

*Each treatment has three replicate populations. Significant differences (P < 0.05) of the mean values between treatments are shown in bold (by t-tests).*

#### Epigenetic Structure

**FIGURE 2:**
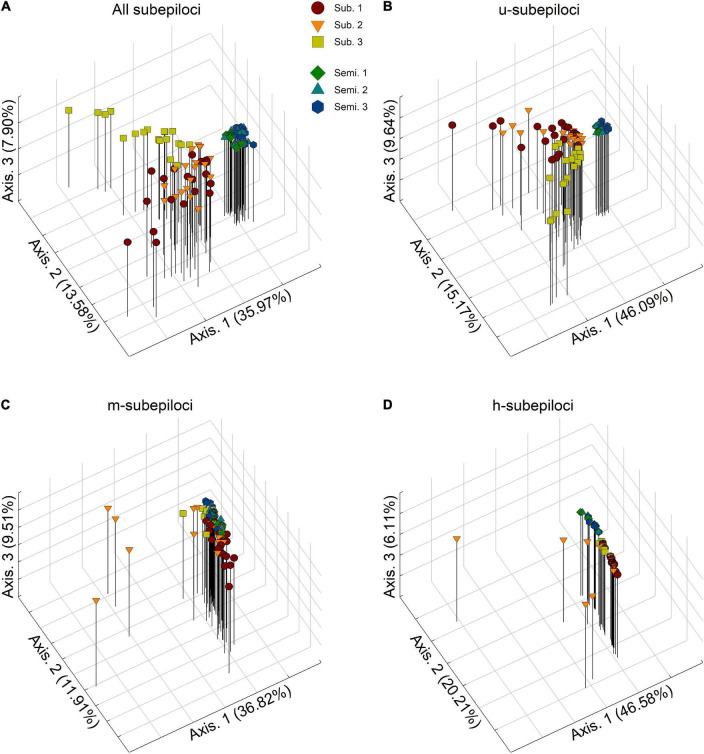
Results of principal coordinate analyses (PCoA) based on **(A)** all subepiloci, and **(B)** u-, **(C)** m-, and **(D)** h-subepiloci of MSAP. Semi. and Sub. stand for the semi-submergence and submergence treatment, respectively.

Principal coordinates analysis revealed that the epigenetic structure of experimental populations differed among different types of subepiloci (Figure 2). Based on all MSAP subepiloci, epigenetic distances separated semi-submergence and submergence treatments along the first axis, forming two separated clusters (Figure 2A). Moreover, the experimental populations 1 and 3 in the submergence treatment fell apart, whereas the experimental populations in the semi-submergence treatment were much closer, with higher convergence degree. A similar differentiation pattern was also found in u-subepiloci, with individuals more clumped in each treatment (Figure 2B). For m-subepiloci, most individuals from different flood regimes grouped together without clear population differentiation, and only some individuals of the submerged experimental population 2 scattered from the cluster (Figure 2C). For h-subepiloci, the two treatments were mainly separated along the second coordinate, forming two clusters, with several individuals separated from the group of the submerged populations (Figure 2D).

For the combined epigenetic dataset (Table 2), AMOVA showed that 36% of epigenetic variance occurred between treatments, 50% within experimental populations, and only 14% among experimental populations within treatments. Similarly, for u-subepiloci and h-subepiloci, most variance occurred between treatments and within experimental populations (for u-subepiloci, 46 and 41%, respectively; for h-subepiloci, 39 and 55%, respectively). However, for m-subepiloci, variation mainly existed within experimental populations (73%).

**TABLE 2:** Results of hierarchical AMOVA based on **(A)** all subepiloci and **(B)** u-, **(C)** m-, and **(D)** h-subepiloci of MSAP.

	Variance	%	ϕ	*P*
**(A) All subepiloci**				
Between treatments	4.131	36	0.363	<0.001
Among populations within treatments	1.568	14	0.216	<0.001
Within populations	5.689	50	0.500	<0.001
**(B) u-subepiloci**				
Between treatments	3.254	46	0.458	<0.001
Among populations within treatments	0.964	14	0.250	<0.001
Within populations	2.895	41	0.593	<0.001
**(C) m-subepiloci**				
Between treatments	0.213	8	0.083	<0.001
Among populations within treatments	0.489	19	0.209	<0.001
Within populations	1.853	73	0.275	<0.001
**(D) h-subepiloci**				
Between treatments	0.664	39	0.386	<0.001
Among populations within treatments	0.114	7	0.108	<0.001
Within populations	0.942	55	0.453	<0.001

#### Outlier Detection

**FIGURE 3:**
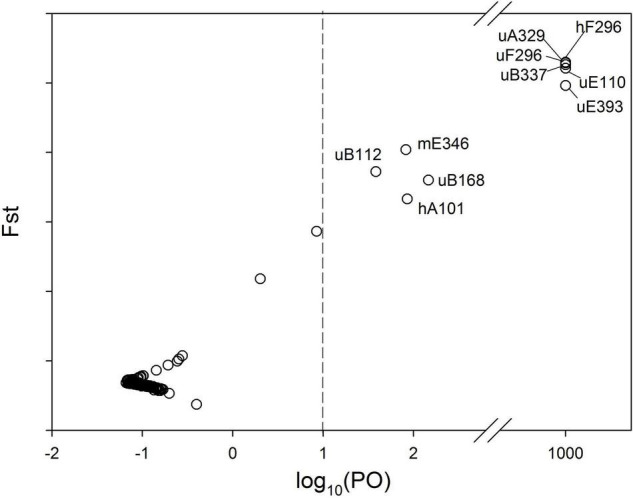
MSAP outliers identified by Bayescan. The posterior odds (PO) for a locus to be under divergent selection are shown on a log scale on the *x*-axis. The vertical dash line indicates the threshold for strong evidence for selection. Outliers on the right side of the vertical line are marked with their names.

For the complete set of the 144 MSAP subepiloci, BayeScan identified 10 (6.9%) outliers (Figure 3), among which seven were u-subepiloci, one was m-subepiloci and two were h-subepiloci, accounting for 10.61, 2.38, and 5.56% of the corresponding type of outliers, respectively. Based on PCoA analysis, outliers clearly separated the semi-submergence and submergence populations along the first axis, while there was no clear differentiation between the two treatments for neutral subepiloci (Supplementary Appendix 4).

### Relationships Among Environmental, Epigenetic and Phenotypic Variation

**FIGURE 4:**
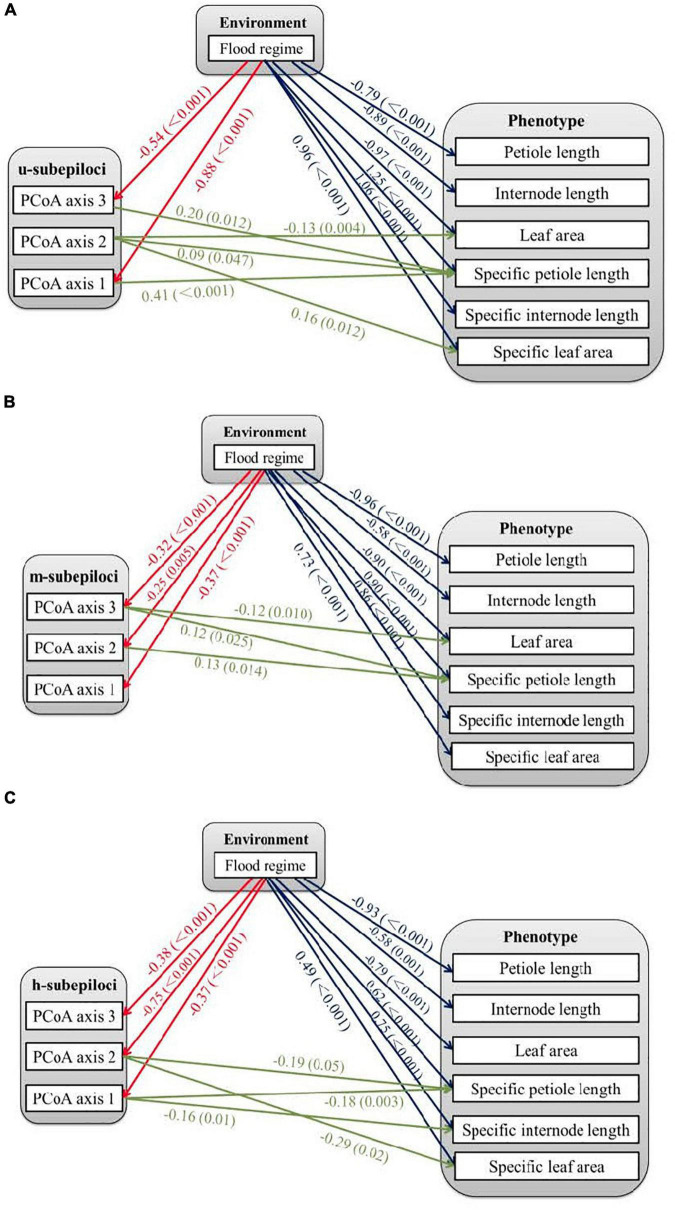
Relationships among environmental, epigenetic and phenotypic variation of *Hydrocotyle vulgaris*. The epigenetic variables are the first three axes from principal coordinate analyses (PCoA) of different types of subepiloci, i.e., **(A)** u-subepiloci, **(B)** m-subepiloci, and **(C)** h-subepiloci. The lines with arrows indicate significant correlations between variables, with coefficients and *P*-values showing near the lines.

The SEM linked the two flood treatments, variation of the different types of subepiloci and the six measured phenotypic traits. The treatments directly affected all traits; however, petiole length and internode length were only significantly correlated with different flood regimes, with no relationship with subepiloci variation (Figure 4). Leaf area and specific leaf area were related to flood-independent u-subepiloci variation (Figure 4), which may arise from spontaneous epimutation. Flood-induced epigenetic variation affected leaf area by m-subepiloci, specific petiole length by all subepiloci types, specific internode length and specific leaf area by h-subepiloci (Figure 4).

## Discussion

### Phenotypic Responses to Different Flood Regimes

Submergence inhibited growth of *H. vulgaris*, possibly due to that decreased irradiance, sediment anoxia, and osmotic stress in this severe environment restrained plant carbohydrate storage, oxygen transport, and nutrient acquisition (Vretare et al., 2001; Santamaría, 2002). However, in response to semi-submergence, *H. vulgaris* may develop flood-tolerant responses and soil-oxygen deficiency resistance, such as elongating stout petiole to extend above the water surface and enlarging thick leaf to capture light and increase gas exchange (Vretare et al., 2001; Luo and Xie, 2009). Moreover, the oxygen transported to the node may drive the length extension of internode for further dispersal (Vretare et al., 2001). These changes could confer a fitness benefit for *H. vulgaris* under semi-submergence, with significant higher aboveground biomass and population density [for ramet aboveground biomass (mean ± SE), submergence = 0.038 ± 0.005 g, semi-submergence = 0.188 ± 0.009 g, *F*_1_, _4_ = 160.791, *P* < 0.001).

### Epigenetic Responses to Different Flood Regimes

Principal coordinate analyses showed a clear epigenetic differentiation between the semi-submergence and the submergence experimental populations of *H. vulgaris*, indicating that environmental conditions could shape DNA methylation patterns of plant populations (Note that if DNA methylation changes largely arise from random epimutation, the presence/absence of private bands would be observed in many loci and such epiloci could be neutral so that the treatment-induced epigenetic differentiation would not occur) (Boyko and Kovalchuk, 2010; Schulz et al., 2014; Zhang et al., 2016). Consistent to previous studies (e.g., Gao et al., 2010; Lira-Medeiros et al., 2010; Li et al., 2013; Zoldoš et al., 2018), our results also suggest that experimental *H. vulgaris* populations can not only respond differently in phenotypic traits, but also undergo a genome-wide epigenetic reprogramming under divergent pressures from contrasting treatments. Therefore, such environment-directed DNA methylation mechanism may be involved in plant adaptation to stress (Boyko and Kovalchuk, 2010; González et al., 2016).

Moreover, the submerged experimental populations of *H. vulgaris* exhibited greater epigenetic diversity and differentiation than the semi-submerged populations. Some of previous studies reported that severe stress could trigger epigenome variability, providing a possible mechanism for fine-tuning short-term adaptive benefits (Boyko et al., 2010; Verhoeven and Preite, 2014; Colicchio et al., 2018). However, long periods of constant stress can fix the allelic variant that confers tolerance to stress *via* strong directional selection, leading to the constrained epigenetic diversity and differentiation (Lira-Medeiros et al., 2010; Grativol et al., 2012; Rico et al., 2014).

Population epigenetic differentiation based on u-subepiloci was highly similar to that based on all MSAP subepiloci. Also, h-subepiloci revealed the semi-submergence and the submergence population cluster. This could indicate a functional difference of subepiloci types, with the additive contribution of u-subepiloci and h-subepiloci to population divergence between the two flood regimes. Moreover, AMOVA results showed that variation mainly existed between treatments and within experimental populations based on both u- and h-subepiloci, similar to that based on all subepiloci, whereas most variation existed only within experimental populations based on m-subepiloci. Therefore, the hemimethylation or demethylation in the CHG-context may play a more important role in habitat adjustment in plants than changes of CG-context. Several previous studies have revealed that u-subepiloci and m-subepiloci are more significant in shaping epigenetic structure of natural populations from different habitats (e.g., Schulz et al., 2014; Zoldoš et al., 2018). Such inconsistency suggests that the function of CG- and CHG-methylated states in response to environmental factors is species- and/or environment-specific (Rico et al., 2014; Putnam et al., 2016). We identified ten outlier epiloci facilitated separation of *H. vulgaris* experimental populations between semi-submergence and submergence (Supplementary Appendixes 4, 5), which may contribute to plastic responses of populations to the flood variation.

### Relationships Among Environmental, Epigenetic and Phenotypic Variation

Structural equation modeling analyses showed that petiole length and internode length of *H. vulgaris* were only significantly correlated with flood, but not with epiloci variation. These results may arise from effects of nutritional or physiological activities, or the low-resolution of MSAP technique (Zhang et al., 2016). Leaf area and specific leaf area are partially affected by u-subepiloci without environmental induction, possibly due to the spontaneous epigenetic variation, arising from imperfect action of enzymes that ensure proper maintenance of epigenetic information through cell division (Verhoeven and Preite, 2014). Stochastic DNA methylation variation is a source for phenotypic diversity in plants, which may mediate phenotypes for several generations that could affect subsequent selection and contribute to adaptive processes (Verhoeven and Preite, 2014; van der Graaf et al., 2015; Groot et al., 2018).

Some phenotypic variation was associated with environment-induced DNA methylation variation through different types of subepiloci, possibly due to their functional differences in regulating gene expression. However, it provides no direct causal information about the region or gene influenced by DNA methylation, as MSAP epiloci are anonymous markers. Our results support the emerging three-way link among flood regimes, DNA methylation and phenotypic changes, suggesting that epigenetic variation might be involved in plastic responses to environmental variation (Wu et al., 2013; Putnam et al., 2016).

## Conclusions

We conclude that plants can exhibit significant phenotypic differences between flood regimes, with clear DNA methylation differentiation associated with phenotypes. Moreover, by using the mixed scoring approach, we find the different contributions of methylation types to epigenetic processes in habitat-related responses. Our study potentially adds to the knowledge base of DNA methylation-environmental interactions. However, we did not demonstrate heritability of the epigenetic changes in later-generation and their long-term adaptive and evolutionary implications. Moreover, information on the mechanistic link between methylation and phenotype is still limited. Therefore, more profound studies are needed to deeply uncover the epigenetic role in plant ecological and evolutionary processes.

## Data Availability Statement

The original contributions presented in the study are included in the article/Supplementary Material, further inquiries can be directed to the corresponding author/s.

## Author Contributions

M-ZW and F-HY designed the research. M-ZW and H-LL performed the research. M-ZW contributed new reagents or analytical tools, analyzed the data, and wrote the manuscript. All authors contributed to the article and approved the submitted version.

## Conflict of Interest

The authors declare that the research was conducted in the absence of any commercial or financial relationships that could be construed as a potential conflict of interest.

## Publisher’s Note

All claims expressed in this article are solely those of the authors and do not necessarily represent those of their affiliated organizations, or those of the publisher, the editors and the reviewers. Any product that may be evaluated in this article, or claim that may be made by its manufacturer, is not guaranteed or endorsed by the publisher.
