# Supplementary material for: DNA Methylation Correlates With Responses of Experimental Hydrocotyle vulgaris Populations to Different Flood Regimes
Source: Front Plant Sci. 2022 Mar 7;13:831175. doi: 10.3389/fpls.2022.831175 (PMC8940293; doi:10.3389/fpls.2022.831175)
Supplement: Supplementary file 1 [file Data_Sheet_1.docx]

**Appendix 1** Scheme of the experimental design. (A) Planting design. The circle represents the soil surface, and the big green dots represent parts of the planted ramets. A total of 91 ramets are evenly planted in the range of a 50-cm-edged hexagon at an interval of 10 cm. To be concise, we only fill one of the six plantation parts of the hexagon in this scheme. (B) Harvesting design. The red triangles represent the 19 sampling points, at an interval of 25 cm.


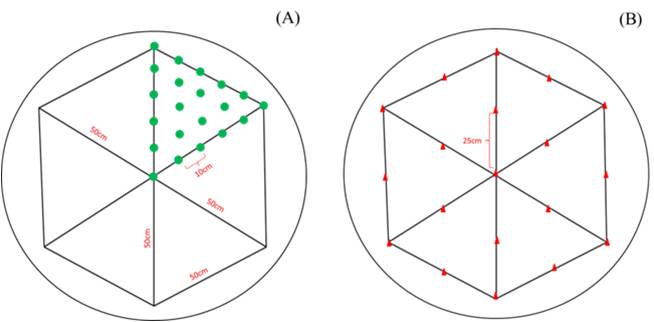


**Appendix 2** Normality test results for six traits

| Traits | Kolmogorov-Smirnova | |  | Shapiro-Wilk | |
| --- | --- | --- | --- | --- | --- |
|  | *K-S* | *P* |  | *W* | *P* |
| Petiole length | 0.306 | 0.084 |  | 0.785 | 0.043 |
| Leaf area* | 0.303 | 0.091 |  | 0.806 | 0.066 |
| Internode length* | 0.201 | 0.200 |  | 0.890 | 0.318 |
| Specific petiole length* | 0.232 | 0.200 |  | 0.850 | 0.159 |
| Specific leaf area | 0.293 | 0.118 |  | 0.842 | 0.136 |
| Specific internode length | 0.314 | 0.064 |  | 0.752 | 0.021 |

Traits with asterisk are log-transformed.

**Appendix 3** Results of *t*-test for epigenetic diversity between the two treatments

| Diversity index | Allsubepiloci | |  | u-subepiloci | |  | m-subepiloci | |  | h-subepiloci | |
| --- | --- | --- | --- | --- | --- | --- | --- | --- | --- | --- | --- |
|  | *t* | *P* |  | *t* | *P* |  | *t* | *P* |  | *t* | *P* |
| Percentage of polymorphic loci (*PLP*) | **-7.339** | **0.002** |  | **-5.928** | **0.004** |  | **-3.718** | **0.021** |  | -1.346 | 0.250 |
| Shannon’s information index (*H*) | **-8.483** | **0.001** |  | **-6.298** | **0.003** |  | **-3.466** | **0.026** |  | -1.408 | 0.232 |

Two-tailed significance (*P*<0.05) are shown in bold.

**Appendix 4** Results of principal coordinates analyses (PCoA) based on outlier and neutral subepiloci.


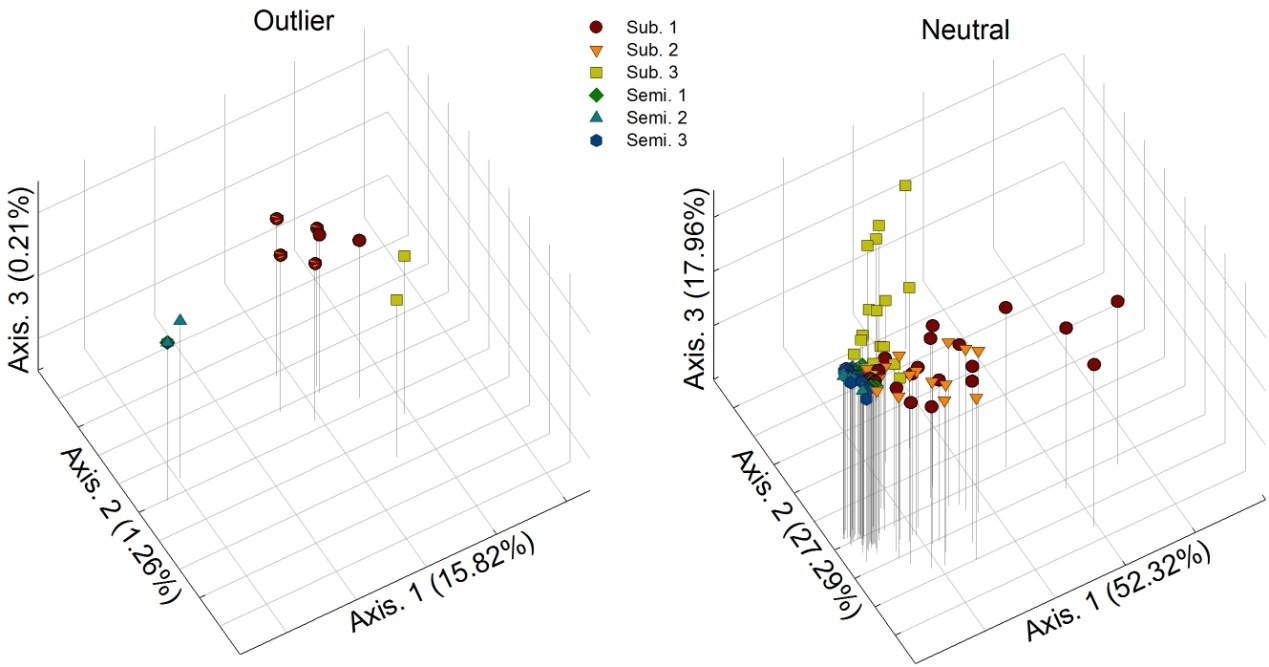


**Appendix 5** Frequency of outliers in each population

|  | uA329 | uB112 | uB168 | uB337 | uE110 | uE393 | uF296 | mE346 | hA101 | hF296 |
| --- | --- | --- | --- | --- | --- | --- | --- | --- | --- | --- |
| Sub. 1 | 19 | 2 | 6 | 19 | 19 | 19 | 0 | 0 | 3 | 19 |
| Sub. 2 | 19 | 0 | 12 | 19 | 19 | 19 | 0 | 0 | 6 | 19 |
| Sub. 3 | 19 | 19 | 19 | 19 | 19 | 19 | 0 | 19 | 17 | 19 |
| Semi. 1 | 0 | 0 | 0 | 0 | 0 | 0 | 19 | 0 | 19 | 0 |
| Semi. 2 | 0 | 0 | 0 | 0 | 0 | 1 | 19 | 0 | 19 | 0 |
| Semi. 3 | 0 | 0 | 0 | 0 | 0 | 0 | 19 | 0 | 19 | 0 |
